# App Chronic Disease Checklist: Protocol to Evaluate Mobile Apps for Chronic Disease Self-Management

**DOI:** 10.2196/resprot.6194

**Published:** 2016-11-04

**Authors:** Kevin Anderson, Oksana Burford, Lynne Emmerton

**Affiliations:** ^1^ School of Pharmacy Curtin University Perth Australia

**Keywords:** health, mobile applications, app, smartphones, self-management, protocol, usability checklist, self-care, chronic disease

## Abstract

**Background:**

The availability of mobile health apps for self-care continues to increase. While little evidence of their clinical impact has been published, there is general agreement among health authorities and authors that consumers’ use of health apps assist in self-management and potentially clinical decision making. A consumer’s sustained engagement with a health app is dependent on the usability and functionality of the app. While numerous studies have attempted to evaluate health apps, there is a paucity of published methods that adequately recognize client experiences in the academic evaluation of apps for chronic conditions.

**Objective:**

This paper reports (1) a protocol to shortlist health apps for academic evaluation, (2) synthesis of a checklist to screen health apps for quality and reliability, and (3) a proposed method to theoretically evaluate usability of health apps, with a view towards identifying one or more apps suitable for clinical assessment.

**Methods:**

A Preferred Reporting Items for Systematic Reviews and Meta-Analyses (PRISMA) flow diagram was developed to guide the selection of the apps to be assessed. The screening checklist was thematically synthesized with reference to recurring constructs in published checklists and related materials for the assessment of health apps. The checklist was evaluated by the authors for face and construct validity. The proposed method for evaluation of health apps required the design of procedures for raters of apps, dummy data entry to test the apps, and analysis of raters’ scores.

**Results:**

The PRISMA flow diagram comprises 5 steps: filtering of duplicate apps; eliminating non-English apps; removing apps requiring purchase, filtering apps not updated within the past year; and separation of apps into their core functionality. The screening checklist to evaluate the selected apps was named the App Chronic Disease Checklist, and comprises 4 sections with 6 questions in each section. The validity check verified classification of, and ambiguity in, wording of questions within constructs. The proposed method to evaluate shortlisted and downloaded apps comprises instructions to attempt set-up of a dummy user profile, and dummy data entry to represent in-range and out-of-range clinical measures simulating a range of user behaviors. A minimum score of 80% by consensus (using the Intraclass Correlation Coefficient) between raters is proposed to identify apps suitable for clinical trials.

**Conclusions:**

The flow diagram allows researchers to shortlist health apps that are potentially suitable for formal evaluation. The evaluation checklist enables quantitative comparison of shortlisted apps based on constructs reported in the literature. The use of multiple raters, and comparison of their scores, is proposed to manage inherent subjectivity in assessing user experiences. Initial trial of the combined protocol is planned for apps pertaining to the self-monitoring of asthma; these results will be reported elsewhere.

## Introduction

Management of chronic conditions has evolved from traditional paper-based monitoring and action plans [1] to the use of mobile messaging [2], and now smartphone and other mobile apps to record and manage clinical data [3-5]. One such application of this technology involved a self-care app for salt intake, which has a protocol published for its use [6]. Although such apps are widely supported by health authorities and authors to enhance consumers’ engagement with self-management, more long-term randomized controlled trials (RCTs) are required to measure their clinical effectiveness and frequency of use [7,8]. Additionally, self-care guidelines should be updated to incorporate engagement with mobile apps during RCTs [9].

Selecting a health app to facilitate self-care of a chronic condition can be overwhelming due to the increasing number of apps for a wide range of health conditions. Engagement with a health app lacking essential operational features, storage and calculation of clinical measures, and unaligned to the consumers’ requirements, can result in declined usage of the app, potentially compromising self-care regimens [10].

Furthermore, many health apps lack a theoretical foundation, as identified in a news post by an emergency room doctor and medical professor in North Carolina [11]. Some apps are structured with a clinical appearance and facilitate data entry by consumers, but are created for entertainment purposes, as acknowledged by another journalist based on the same doctor’s findings [12]. Additionally, consumers’ decisions to select apps presented in the Apple App Store and the Google Play Store are clouded by marketing jargon and lay-user reviews, with an absence of official and consistent quality markers [13].

The certification of health apps to improve safety and quality in health care is an ongoing issue [14]; theory-based quality ranking of apps has begun [15] but is in an early stage. Proposed interventions include active review of every health app by app stores and/or regulators such as the Food and Drugs Administration (FDA) in the United States or the Therapeutic Goods Administration (TGA) in Australia [14]. This method is expected to be relatively slow and costly. Complicating this problem, many health apps do not fall within the jurisdiction of the FDA [5], TGA, or their overseas counterparts, particularly if the apps are not classified as medical devices and have no peripheral device requiring regulatory assessment. Consequently, the need for further research into the clinical integrity of health apps is warranted.

A recently published initiative using a rating scale for health apps named the Mobile Application Rating Scale (MARS) [16] was produced in Australia, and designed to aid app selection by researchers. The MARS appears comprehensive when rating mental health and general health apps, but has not been specifically designed for chronic conditions. Additionally, the 23 sub-categories of the MARS were not all grounded in health consumer mobile app experiences; some usability studies informing the MARS included health website evaluation [17], nonhealth website quality measurement [18,19], user experiences with online goods [20], and nonhealth-specific evaluation frameworks [21]. One recent study questioned the MARS’ validity, since it has not been widely adopted [22]. However, building or updating an app to rate against the MARS requires due process, and more findings are expected since an Australian state government healthy body endorsed the scale, attracting media attention [23].

A number of other studies regarding the usability of health apps have reported findings [24-26], a content analysis guide [27], a mobile website framework [28], and an app design and development guideline [9]. One app-usability study [28] built upon Nielsen’s usability heuristics [29], but was not health-tailored. Table 1 outlines health app usability studies that have produced checklists or rating scales; these are critiqued later in this paper. Growth in the health app market, both in terms of availability and adoption, warrants greater distinction between apps. A need exists for a protocol to guide researchers in their identification of apps suitable for assessment, and for developers to test their product against competitors’ apps. This paper reports (1) a protocol to identify relevant apps for academic evaluation, (2) synthesis of a checklist to screen apps for quality and reliability, and (3) a proposed method to theoretically evaluate the usability of health apps, with a view towards identifying one or more apps that are suitable for clinical assessment.

**Table 1:** Commonalities and differences between health app usability studies.

Authors	Year	Name of rating scale or checklist	Purpose	Consumer vs academic use	Number of dimensions	Number of raters
Stoyanov et al [16]	2015	Rating scale^a^: Mobile Application Rating Scale (MARS)	Quality assessment	Academic	5	2
Nielsen [29]	1994	Checklist^b^: Nielsen’s Usability Heuristics	Rectify usability problems	Academic	10	3-5
Hundert et al [5]	2014	Checklist: 7 criteria	Headache diary app evaluation (scored against 7 criteria)	Both help to inform health care professionals and potential users on the best available e-diary apps for headaches	7	2
Belmon et al [30]	2015	Rating scale: for app features, not complete apps; Behavior Change Techniques (BCT)	Young adults’ opinion on BCT in physical activity apps	Consumer rating	3	N/A (179 young Dutch adults)
Patel et al [15]	2015	Rating scale: MARS [16]; (1) Weight loss/smoking cessation criterion score, (2) cultural appropriateness criterion score, and (3) cultural appropriateness criteria	Quality ranking	Academic	3 with 22, 23, and 6 sub-criteria, respectively	2
Yanez Gomez et al [31]	2014	Mobile-specific usability heuristic checklist	Heuristic evaluation	Academic	13	As per Nielsen [29]

^a^A rating scale’s results align a numerical value to constructs such as *Ease of Use*.

^b^A checklist can be a series of requirements necessary to achieve compliance without numerical values.

## Methods

### Phase 1: Development of an App Selection Protocol

Selection of relevant apps (and elimination of irrelevant apps) requires sequential consideration of the publicized and evident features of apps. A Preferred Reporting Items for Systematic Reviews and Meta-Analyses (PRISMA) flow diagram was deemed suitable for representation of the shortlisting process. In the absence of guidance from published literature, critical decisions for the purposes of shortlisting health apps were based on:

Relevance: limiting searches to the respective country’s app stores ensures relevance to the local setting. Duplicate apps require removal from the shortlist. Preliminary trial of the PRISMA flow diagram has identified some apps available on both iOS and Android operating systems with similar names, requiring further examination of app logos and *screen dumps* available from the respective app store. Cases in which both an Apple and Android version of an app are available result in the Apple version being recommended to be retained, since health apps with clinical management in Australia are launched on iOS first (Brophy S, personal communication, 1 January 2015).

Availability in English: this enables evaluation of the app in the local environment. Preliminary trial of the selection process has indicated that some apps displayed in a language other than English are also available in English once the app has been downloaded.

Provision of clinical management: preliminary trial of the flow diagram suggests health apps can be classified into 5 categories. *Clinical management apps* require the user to input clinical readings such as peak expiratory flow (for asthma monitoring) or blood pressure (for hypertension monitoring), and may integrate gamification for sustained usage of the app. *Informational apps* or *eBooks* are simply digitized books containing information about a condition, without facilitating data input. *First aid apps*, ambulance apps or individual doctors’ apps were classed as extraneous to the use of the app for self-monitoring of a medical condition. *Exercise or yoga apps* involve holistic management of the medical condition through techniques such as controlled breathing techniques or yoga poses. *Novelty apps* or apps for entertainment purposes include prank apps and games using fictional characters with the target condition. Certain apps, identified through searches restricted to Australia, are only available via an international account, and have been categorized accordingly.

Availability at no cost to consumers: if the purpose of the shortlisting and evaluating apps is to identify an app(s) suitable for formal evaluation via clinical trial, or as part of the outcome measures in a trial, ideally the app(s) should be available at no cost to consumers. This parameter assumes that the cost of an app is unrelated to quality of the app.

Currency: the date of the most recent update is a particularly important eligibility criterion, since it represents the frequency with which developers respond to consumer feedback.

### Phase 2: Development of the Evaluation Checklist

The app evaluation checklist was synthesized using peer-reviewed checklists and studies on the usability of health apps [5,15,16,25,27,29-35], supplemented with a qualitative study exploring consumer experiences with health apps [10]. Critique and comparison of the extant checklists, and the proposed checklist, are presented in the *Results* section. Criteria-based quality assessment was applied by creating the checklist in a number of iterations, data reduction [36,37], and assessment of face and construct validity by the authors. Face validity involved reviewing syntax and structure of checklist questions to ensure that questions reflect the research objectives. Construct validity required testing the definition of themes; these discrepancies were verified using definitions provided by similar studies, and cross-referenced with theoretical models.

This checklist was also created with reference to the principles of heuristic evaluation [29,38], which encompasses the construction of small but broad *usability principles* to evaluate an app’s usability [29]. Heuristic evaluation has been applied successfully in the development of a number of health apps, such as headache diaries [5] and healthy eating apps [39], to guide design features such as the maximum number of items to maintain comprehensiveness, specificity, and efficiency. Nielsen’s Usability Heuristics [29] were the foundation of several mobile app usability studies [5,28,31], and were applied here. The checklist was designed to enable rating by assessors, as per another Australian health app study [16]. For efficiency and to avoid transcription errors, the checklist should be created with survey software such as Qualtrics, rather than in hard copy.

Heuristic evaluation involved the application of 10 principles to each app, as reported by the Oracle Corporation [38]:

1. Visibility of system feedback: can the system show the user what part of the system is being accessed? Does the *back* button inform the user where they are returning to?

2. Complexity of the application: is the information technology and health literacy displayed in the app applicable to the target audience?

3. Task navigation and user controls: is the shortest possible path taken for users to perform tasks?

4. Consistency and standards: are industry standards adhered to, so users are not confused about the meaning of certain standards (eg, metric units) or conventions?

5. Error prevention and correction: are users prevented from making errors, such as entering letters in a numbers field?

6. Recognition rather than memory overload: does the system help people remember, rather than presenting all information at once?

7. Efficient to use: is there a basic and advanced mode to cater to different users?

8. Simplicity and appeal: is the system and design easy to use/appealing?

9. Be tolerant and reduce cost of errors: do errors provide avenues for further support? Can users move on after an error?

10. Help support: are there helpful suggestions for users to follow when unsure how to proceed?

### Phase 3: Development of the Method to Evaluate the Usability of Health Apps

In order to apply the evaluation checklist to selected apps, a number of procedures are required: (1) determination of the number of independent raters; (2) moderation of differences between raters; (3) instructions for set-up and simulated use of the app, such as identification of a realistic user profile for all raters to enter; (4) standardization of time for initial navigation of the app; and (5) particular tasks to attempt to represent a range of user behaviors, and test the limits of the app. A simple summative scoring system is suggested to identify those apps considered to have met the criteria for formal evaluation or inclusion in a clinical trial. The scores of multiple expert raters should be compared using the 2-way mixed Intraclass Correlation Coefficient (ICC), since the same raters rate shortlisted apps using the same checklist. Consideration of interrater reliability using the ICC with SPSS version 23 (IBM Corp., Armonk, NY; 2015) is used. Utilization of the ICC is recommended to capture the varying *magnitudes of disagreement* [5] present in subjective usability metrics, and to measure homogeneity amongst raters. Internal consistency should be assessed using Cronbach alpha to ensure questions used in each section of the questionnaire are measuring the same construct [5,40]. Instructions for management of these calculations are presented in the *Results* section.

## Results

### Phase 1: Development of an App Selection Protocol

**Figure 1:**
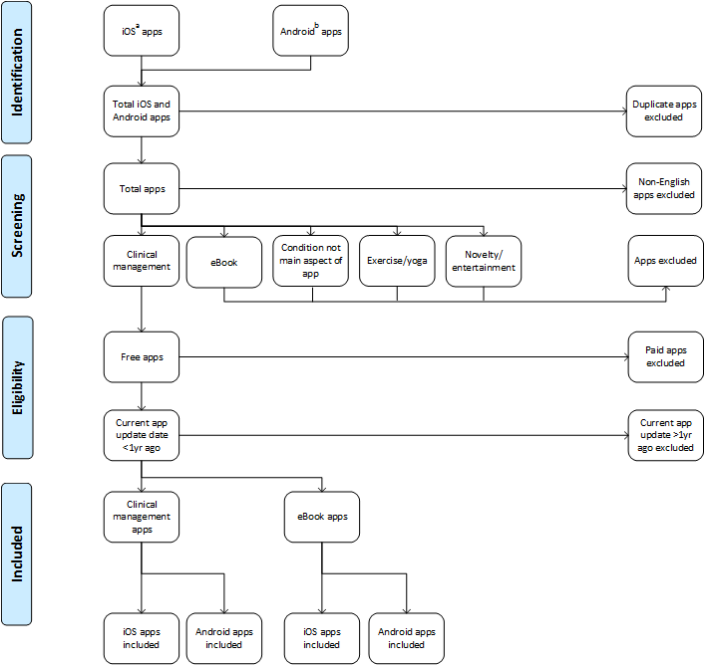
PRISMA flow diagram.^a^Via Australian iOS APP Store (iTunes),^b^Via play.google.com (Australian account).

The process for filtering health apps available from the Australian Apple App Store and the Google Play Store to meet selection criteria is represented in Figure 1. In line with the 5 critical decisions described in the *Methods*, the flow diagram assesses relevance, English language, clinical management, free availability, and currency of the version.

This app-identification procedure uses the Australian Apple App Store and Google Play Store to locate apps specific to the target chronic condition. Subsequently, duplicate apps are removed, in addition to foreign language apps with no English language option. Apps not providing clinical management of the target condition are removed. Only free apps that have been updated less than 1 year ago are retained.

### Phase 2: Development of the Evaluation Checklist

In total, 6 peer-reviewed checklists focusing on usability of health apps were identified [5,15,16,29-31], as presented in Table 1. The MARS comprises 4 dimensions, totaling 19 items, with another subjective quality and app-specific category of 10 items [16]. Dimensions used in the 6 studies ranged from 3 to 13. Overall, there were consistent themes in the extant checklists, but subcomponents (ie, warnings about unhealthy values, user profile setup, and features available in offline mode) were lacking.

In addition to the studies described in Table 1, 1 app usability framework for health websites provided useful insight into theory underlying the Technology Acceptance Model (TAM) and user experience [28]. Another study [41] was not health related, but guided creation of the checklist, with reference to some common considerations regarding app usability, such as design and *help* features. Self-care guidelines when using an app were also instrumental in guiding the design of this protocol, although no rating scale or checklist were evident [9]. One content analysis guide for smoking apps [27] confirmed findings from the aforementioned studies including feedback, app content, user relevance, and user experience.

Other peer-reviewed studies have reported health app usability research without applying checklists, rating scales, guidelines, or frameworks. A New Zealand ranking system for weight loss and smoking cessation apps used 22 and 23 items respectively, considering social networking synchronization, daily activities (eg, record of food intake), personalized feedback and engagement, and using a Boolean operator to award points for scoring purposes [15]. The items listed in this New Zealand study were specific to the health condition, rather than considering other factors affecting app quality. Additionally, 2 studies presented methods to select the most popular apps to rate [15,27], rather than create a checklist or rating scale for comparative assessment of apps. Comparing and contrasting the aforementioned checklists confirmed the need for the design process to consider how consumers maintain self-care practices.

Table 2 lists the constructs, variables, and source(s) of each variable in the resultant checklist, named the App Chronic Disease Checklist (ACDC); the complete checklist is illustrated in Multimedia Appendix 1. In total, 4 constructs (*Engagement*, *Functionality*, *Ease of Use*, and *Information Management*), derived from thematic analyses of published checklists and qualitative research, are represented in the checklist. A qualitative study [10] informed the need to include *Ease of Use* as a construct (rather than *Aesthetics*, a theme from the MARS), and broaden the scope of the *Information Management* construct.

**Table 2:** Thematic synthesis of the ACDC checklist.

Construct	Variable	Source
Engagement	Gamification	[10,15,42]
	Customization	[10,16,33,43]
	Interactivity	[10,16]
	Positive Behavior Change	[10]
	Effectiveness	[16]
	Self-Awareness	[10,16,30]
Functionality	Health Warning	[10]
	Feedback	[10,16,27,29,31,34,39,44]
	Intuitive Design	[10,16,33,34]
	Connection to Services	[10,16,24]
	Performance Power	[10,16,29]
	Structural Navigation	[16,29,31]
Ease of Use	Usability	[10,16]
	Automation	[10,26]
	Medical and Technological Jargon	[10,39]
	User Profile Setup	[10]
	Offline Mode	[10]
	Reminders	[5]
Information Management	Statistics	[5,10]
	Privacy and Data Security	[10,43-46]
	Quality and Accuracy of Information	[10,29,34,39,46]
	Quantity of Information	[16,39]
	Visual Information	[10,16]
	Credibility	[16]

Face and construct validity were confirmed via discussion amongst the 3 authors. Construct validity guided the classification of, and ambiguity in, wording of questions within constructs, as guided by the TAM [47] and Health Information TAM [48]. The TAM confirmed alignment of questions relating to *Reminders* and *Automation* within the *Ease of Use* construct. This process was undertaken simultaneously with the consideration of usability heuristics. Lack of information in studies considering *Visual Appeal*, for example, was addressed by using Nielsen’s Usability Heuristics [29] and integrated into the *Functionality: Feedback* and *Information Management: Visual Information* questions. Discussion amongst authors and consideration of extant checklists determined that a 3-point ordinal scale, appropriately worded for each question, would be used. Details of this scoring scale are described later in this paper.

### Phase 3: Development of the Method to Evaluate the Usability of Health Apps

The evaluation should be completed as soon as possible after shortlisting of apps, to ensure version control and currency. In two studies, 2 raters were used to apply scores to apps [5,16], while 1 study used 5 raters to measure usability [9]. This approach was consistent with the recommendation by Nielsen [29] to use 3 to 5 experts. In line with these recommendations, and a number of other health app studies [5,16,41], this protocol suggests 3 expert raters with no experience or conflicts of interest with any of the apps.

All clinical management apps retained by the flow diagram should be rated without collusion between raters, and in their entirety, before proceeding to a subsequent app. Initially, a sample (approximately 10%) of these apps should be randomly identified using a randomization algorithm, and quarantined for trial scoring by all raters, with results being moderated between the raters. Scores from this trial may be merged into the full scoring exercise if no significant changes have been made to the scoring protocol, as recommended by methodologists [40]. If a trialed app and a nontrialed app produce the 2 top scores, both scores should be moderated to identify the top-ranked app.

After proceeding with the assessment of the remaining shortlisted apps, raters’ scores (saved in the online survey platform) will be imported to SPSS for calculation of usability scores and interrater and internal reliability. Each response on the 3-point ordinal scale will be assigned a value of 0 (where the feature is not evident or functional), 0.5 (where the feature is somewhat evident or functional), or 1 point (where the feature is clearly evident or functional), and summed to a total (out of 6) for each of the 4 constructs, as well as a total out of 24 for each app.

As established in the *Methods*, 2-way mixed ICC is recommended to measure interrater reliability [49]. The ICC should be calculated for the total score (out of 24) to compare the 3 raters, and the raters’ totals for each construct: *Engagement*, *Functionality*, *Ease of Use*, and *Information Management*. Differences in scores should only warrant moderation if the ICC for each construct is nonsignificant (*P*>.05). Subjective questions, such as those within the *Ease of Use* construct, are expected to generate a lower ICC score in that construct, compared to more objective ratings of items relating to *Privacy* or *Ability to Export Data*.

One Cronbach alpha statistic should be calculated to measure correlation between the collective totals for each construct (out of 18 for each construct, if using 3 raters). Cronbach alpha should also be determined for the total score (out of 72) for the 3 raters collectively.

Before the apps are set up, instructions commence by entering all remaining shortlisted apps into a random list generator. The purpose of randomizing apps is to eliminate selection bias by balancing *unknown factors* [50]. Apple HealthKit apps actively monitor consumer readings, so raters should create unique logins that are clearly identified as being associated with trial of the app (eg, a consumer name such as *Test Dummy 1*); however, raters should provide authentic contact details for compulsory profile fields to facilitate receipt of outputs, if this is a function of the app. If raters encounter requests for additional data, the recommended approach is to refer to the Instructions for Raters (Multimedia Appendix 2).

**Figure 2:**
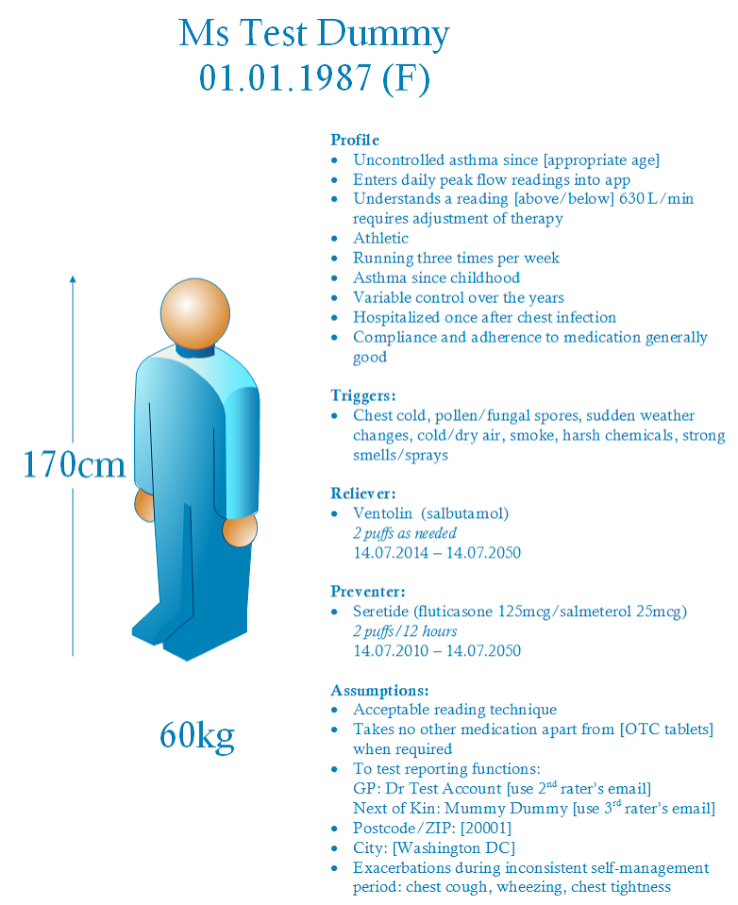
Test dummy profile for clinical data entry.

Figure 2 illustrates the features of a dummy profile for entering clinical data into shortlisted apps to gauge the app’s usability and functionality. The dummy profile comprises a range of realistic goals, and demographic and clinical data that reflect information that might be requested of new users. These data should be adjusted by the lead investigator to be realistic for the medical condition of interest (eg, obesity management).

**Figure 3:**
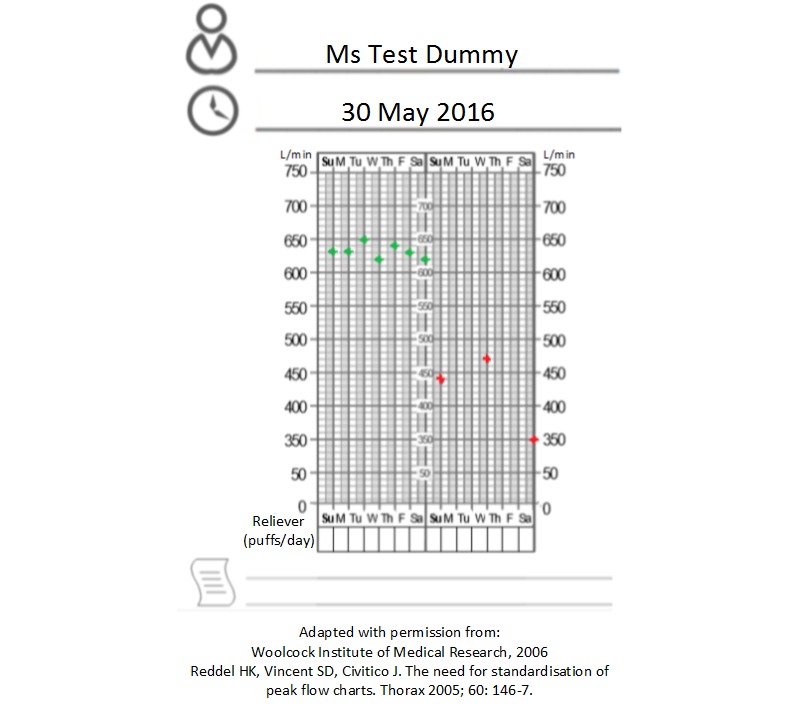
Peak flow values to input into shortlisted clinical management apps.

As part of the dummy profile, raters should attempt to enter 1 week of realistic in-range clinical readings, taken with good compliance, with the recommended self-monitoring schedule for the relevant medical condition. This week should be followed by 1 week of readings representing poor control of the medical condition, with several days of poor compliance with self-monitoring. An example based on peak expiratory flow readings (for asthma monitoring) is provided in Figure 3, in which an adverse event such as a respiratory infection (in red) has affected a consumer’s readings, and numerous readings are missing during this period of out-of-range data. Such variations in clinical data are important to gauge how the clinical management app responds to variable control of one’s chronic condition and inconsistency in data entry. If raters encounter requests for additional data, the recommended approach is to discuss a course of action with other raters before proceeding.

## Discussion

Creating a health app selection protocol for developers and academics resulted in a guided and evidence-based procedure that aims to guide researchers to identify a health app with the highest level of usability and functionality characteristics. The identified app may then be the subject of a clinical trial as an independent intervention in health consumers’ self-management of a chronic condition, or as an adjunct for other interventions. The need for evidence-based content when deciding which health app to use is also supported by a 2016 Australian review of mental health apps [51]. Consequently, consumers using top-ranking apps identified by this protocol are expected to demonstrate greater persistence with self-management of medical conditions. This theory, however, remains to be tested.

Dissemination of this protocol should also benefit app developers in their appreciation of usability heuristics and features of highly-functional, high-quality, and attractive apps. Future variations could include a developer-specific checklist, with design science and computer science-related constructs aiding the app design and development process.

The key contribution of this protocol to the body of research in this field lies in its comprehensiveness. This protocol incorporates a 3-stage method to shortlist apps, and then assesses the shortlisted apps using standardized instructions for a team of raters using an evidence-based checklist (the ACDC). The use of 3 expert raters is expected to be economical, without compromising robustness; trial of the protocol and determination of the interrater reliability statistics are required to confirm this theory.

While a previous study reported a brief flow diagram for the selection of an app [16], the inclusion of more selection criteria in the flow diagram enables more discriminatory filtering of available apps. The number of apps retained by this filtering process is expected to vary according to the chronic condition and number of marketed apps. Additional shortlisting criteria may be included if the final number retained apps remains unmanageable.

The ACDC draws most heavily on the MARS [16], with a number of differences informed from the review of other literature, and recognizes that findings from the MARS have not yet been published. First, *Ease of Use* has been identified as a construct in the ACDC, rather than *Aesthetics* (in the MARS). This development was informed by qualitative research [10] that reported strong consumer sentiment in health app experiences. By including this consumer perspective, the ACDC recognizes the importance of a consumer’s persistence with a health app for self-management of a chronic condition [10,52,53]. Second, the *Information Management* construct has been broadened in the ACDC to reflect data concerns in the information age, as informed by qualitative research [10]. Third, the ACDC was designed for use in apps for any chronic condition, not just mental health, which is the reported use for the MARS [16]. Fourth, a limitation of the MARS identified in the *Introduction* was the MARS’s construction with reference to sources beyond health app usability studies. The ACDC was constructed via thematic synthesis from a body of literature specific to health app usability.

Apps are being launched with increasing frequency, and considering the ubiquitous nature of smartphones and electronic health strategies of hospitals and clinics, the use of health apps to facilitate self-care of chronic conditions will continue to expand. The authors acknowledge the release of Apple’s ResearchKit [54] and the more individualized CareKit [55], which harbor the ability of researchers to embed surveys in Apple apps for data reporting. Android-based smartphones will soon have access to these open-source Apple apps (eg, Asthma Health [56]) that are available for American Apple account holders only. In the future, authors of clinical outcome questionnaires should enable researchers to integrate questions into platforms such as ResearchKit, for efficiency and convenience of data entry during clinical trials.

It is essential for developers and academics to employ a profile with dummy values to test the shortlisted apps, with the profile including compliant and noncompliant clinical readings, in addition to registering a real email account to which readings can be exported. One limitation of this approach is that a single dummy profile, even devised with in-range and out-of-range clinical data, is unlikely to test the full functionality of an app. However, a carefully constructed dummy profile and the use of 3 raters, each completing a 24-question assessment of the app, should enable thorough evaluation and ranking of the shortlisted apps.

This protocol offers a comprehensive procedure and straightforward checklist to guide selection of highly-functional and usable health apps for use in further research, or self-management by consumers. To date, the protocol has been partially tested; the first research study will apply this protocol to apps for asthma self-management.

## Appendix

Multimedia Appendix 1 App Chronic Disease Checklist v1.0.

Multimedia Appendix 2 Instructions for raters.

## Abbreviations

ACDC: App Chronic Disease Checklist
BCT: Behavior Change Technique
FDA: Food and Drugs Administration
ICC: Intraclass Correlation Coefficient
MARS: Mobile Application Rating Scale
PRISMA: Preferred Reporting Items for Systematic Reviews and Meta-Analyses
RCT: randomized controlled trial
TAM: Technology Acceptance Model
TGA: Therapeutic Goods Administration
