# Supplementary material for: App Chronic Disease Checklist: Protocol to Evaluate Mobile Apps for Chronic Disease Self-Management
Source: JMIR Res Protoc. 2016 Nov 4;5(4):e204. doi: 10.2196/resprot.6194 (PMC5116100; doi:10.2196/resprot.6194)
Supplement: Multimedia Appendix 1 [file resprot_v5i4e204_app1.pdf]

Multimedia Appendix 1

App Chronic Disease Checklist v1.0

Q1.1 What is your name?
- ❍ Rater 1
- ❍ Rater 2
- ❍ Rater 3
- ❍ Group Consensus
- ❍ Dummy Values for Protocol

Q1.2 What is the name of the app you're rating?

____

Q1.3 On which platform are you using this app?
- ❍ iOS
- ❍ Android

Q1.4 Which chronic condition does this app address?
- ❍ Asthma
- ❍ Hypertension
- ❍ Mental Health
- ❍ Other

ENGAGEMENT

Q2.1 Gamification: Does the app apply gaming principles (such as digital rewards, prizes, leaderboards, badges, aggregated readings or competitions) to engage users?

❍ No, or not apparent initially (for new users)
❍ Yes - one/some
❍ Yes - numerous

Q2.2 Customisation: Does the app allow customisation of features (e.g. sound, content, notifications etc.)?

❍ No, app does not allow any customisation
❍ Yes - but limited, e.g. requires resetting each time
❍ Yes - allows comprehensive customisation

Q2.3 Interactivity: Does the app enable users to enter free-text reflections alongside their clinical data?

❍ No, or not apparently
❍ Yes - but limited
❍ Yes - ample free-text options

Q2.4 Engagement through Use of Plug-ins: Can the app connect with a peripheral device (e.g. via Bluetooth)?

❍ No, or not apparently
❍ Yes - but not obvious, or not a key feature of the app
❍ Yes - obvious and a key feature of the app

Q2.5 Self-Awareness: Does the app encourage the user to develop self-reflection and/or increased self-awareness of the chronic condition?

❍ No, or not apparently
❍ Yes - but to a limited extent
❍ Yes - comprehensively

Q2.6 Positive Behavior Change: Does the app encourage positive self-care practices (lifestyle or behavioural action), e.g. using reminders, tips or social influences?

❍ No, or not apparently
❍ Yes - but to a limited extent
❍ Yes - comprehensively

FUNCTIONALITY

Q3.1 Health Warnings: Does the app provide warnings about, or highlight, out-of-range readings?
❍ No warnings or highlights provided
❍ Yes, warnings/highlights provided, but with no guidance/support
❍ Yes, warnings/highlights provided, with guidance/support

Q3.2 Feedback: Does the app provide tactile, visual and/or sound feedback?
❍ No tactile, visual and/or sound feedback provided
❍ Limited tactile, visual and/or sound feedback provided
❍ Comprehensive tactile, visual and/or sound feedback, with additional controls/features (e.g. on/off, calendar integration)

Q3.3 Structural Navigation: Does the app facilitate sequential/appropriate navigation?
❍ No, or not apparently
❍ Yes, but with some deficiencies in functionality/links
❍ Yes, seamless structural navigation

Q3.4 Intuitive Design: Is the app designed for intuitive use (e.g. identifiable data input fields, intuitive symbols, generous touch areas)?
❍ No, or not apparently
❍ Yes, but to a limited extent
❍ Yes - comprehensively

Q3.5 Connection to Services: Does the app have capacity to send or connect data to another service (e.g. Apple Health)?
❍ No, or not apparently
❍ Yes - but to a limited extent (e.g. email data to self only)
❍ Yes - comprehensively

Q3.6 Performance Power: How fast do the app features (functions) and components (buttons/menus) work?
❍ Slow or inefficient, times out or crashes
❍ Reasonably efficient
❍ Very efficient, additional functionality (e.g. ‘loading time’ indicator)

EASE OF USE

Q4.1 Holistic Usability: Can all relevant self-management tasks be easily completed in this single app?

- ❍ No - not easily; requires another app to record certain/limited instructions
- ❍ Yes - but still requires another app
- ❍ Yes - all readings can be recorded on this app

Q4.2 Automation: Does the app facilitate automation of tasks, e.g. with pre-populated fields, suggestions based on inputs, management of medical appointments, automated customer service?

- ❍ No, or not evidently
- ❍ Yes - limited automation evident
- ❍ Yes - comprehensive automation evident

Q4.3 Medical and Technological Jargon: Is the app free from confusing (medical and/or technology) jargon?

- ❍ No - jargon evident
- ❍ Yes - mostly consumer-friendly terminology
- ❍ Yes - consumer-friendly terminology throughout

Q4.4 User Profile Setup: Does the app provide easy setup of a user profile, e.g. option to login via social media account?

- ❍ No, app operates without user profile or registration upon download
- ❍ Yes - limited user profile setup present
- ❍ Yes - easy, guided, comprehensive profile; social media login provided

Q4.5 Offline mode: Does the app operate in offline mode?

- ❍ No - app does not operate in offline mode
- ❍ Yes - but limited offline functionality
- ❍ Yes - comprehensive features available in offline mode; syncs when back online

Q4.6 Reminders: Does the app enable users to set reminders?

- ❍ No, or not apparently
- ❍ Yes - basic reminders can be set
- ❍ Yes - advanced reminders can be set, e.g. synced with external calendar, integrated with SMS

INFORMATION MANAGEMENT

Q5.1 Statistics: Does the app enable analysis of clinical data (e.g. produces statistics, graphs)?
- ❍ No, or not apparently
- ❍ Yes - but limited analysis
- ❍ Yes - comprehensive statistics available; can be exported for further analysis

Q5.2 Privacy and data security: Does the app allow secure data input and export (e.g. password management, encryption, privacy statement, cloud backup)?
- ❍ No, or not apparently
- ❍ Yes - basic security/privacy features
- ❍ Yes - comprehensive security/privacy features

Q5.3 Quality and Accurate Information: Does the app accept and display correct, relevant information regarding the chronic condition?
- ❍ No, or not apparently
- ❍ Yes - but with some limitations, e.g. does not detect out-of-range values entered
- ❍ Yes - comprehensive controls over data entry; entered data consistent with displayed outputs

Q5.4 Quantity of information: Is health information offered by the app concise but still comprehensive?
- ❍ No, too minimal or overwhelming
- ❍ Yes - but gaps or unnecessary detail present
- ❍ Yes - offers concise but comprehensive information

Q5.5 Visual information: Is visual explanation of concepts – through charts, graphs, images, videos etc – clear, logical and correct?
- ❍ No - unclear, illogical or incorrect visual information
- ❍ Yes - but sometimes unclear, illogical or incorrect
- ❍ Yes - clear, logical and correct

Q5.6 Credibility: Is the app developer/producer credible, e.g. uses a recognised logo or cites research?
- ❍ No, or not apparently, e.g. no credentials displayed
- ❍ Yes - credentials displayed
- ❍ Yes - comprehensive credentials displayed; cites research
