# Supplementary material for: App Chronic Disease Checklist: Protocol to Evaluate Mobile Apps for Chronic Disease Self-Management
Source: JMIR Res Protoc. 2016 Nov 4;5(4):e204. doi: 10.2196/resprot.6194 (PMC5116100; doi:10.2196/resprot.6194)
Supplement: Multimedia Appendix 2 [file resprot_v5i4e204_app2.pdf]

Multimedia Appendix 2. Instructions for raters.

Step	Preparation tasks	Required Kit	ACDC Reference
**1**	Assemble the list of apps retained by the PRISMA flow diagram<br>Use www.random.org to select which apps all raters are to assess in the same order (thus minimising the risk of updates influencing scores)	Retained app names<br>www.random.org	
**2**	Install each clinical management app from respective app store	Mobile device with internet connection, completed PRISMA flow diagram to determine which app	
**3**	Follow displayed instructions for new users such as establishing or registering a user profile	Smartphone with internet connection	Ease of Use: User Profile Setup
**4**	Use dummy profile data in Figure 2 as created by own lead investigator and insert clinical values	Smartphone with internet connection, dummy profile data (Figure 2)	
**5**	Spend five minutes familiarizing yourself with the app<br>Close the app completely then re-open it	Smartphone with internet connection	
**Observations during Clinical Data Entry**			
	When performing clinical data entry below:		
**6**	Note the visual appeal of the user interface	Refer to Step 3	Functionality: Graphics and Visual Appeal
**7**	Identify sequential, structural navigation	Refer to Step 3	Functionality: Structural Navigation
**8**	Recall responsiveness of app features and components	Refer to Step 3	Functionality: Performance Power
**9**	Observe medical or technical jargon	Refer to Step 3	Ease of Use: Medical and Technical Jargon
**Clinical data entry**			
**10**	Enter clinical measures such as peak flow readings for one week using healthy range values as per Figure 3	Lead investigator to input dummy profile and set of readings similar to Figure 2 + 3	Functionality: Intuitive Design<br><br>Information Management: Quality and Accurate Information
**11**	Enter clinical measures (as appropriate) for one week using uncontrolled and unhealthy range values and some missing values (Figure 3 is a guide)	As above	Functionality: Intuitive Design<br><br>Information

			Management:<br>Quality and Accurate Information
**App functionality permitting, complete the following tasks**			
**12**	Note any health warnings provided when inputting unhealthy range values	Refer to Step 3	Functionality:<br>Health Warnings
**13**	Respond to feedback provided by app such as personal strengths or limitations	Refer to Step 3	Engagement:<br>Self-Awareness
**14**	View graphical representation of inputted data in graph and numerical form	Refer to Step 3	Information Management:<br>Visual Information
**15**	Add notes to data entries e.g. high blood pressure due to weightlifting class	Refer to Step 3	Engagement:<br>Interactivity
**16**	Email the data or outputs to yourself and another rater<br>Ensure emailed data (e.g. .xls, .csv) is readable without significant formatting required	Smartphone mail configuration	Functionality:<br>Connection to Services
**17**	Access points/rewards system – have your readings accrued any points?	Refer to Step 3	Engagement:<br>Gamification
**18**	Add goals<br>(lead investigator should devise appropriate goal(s) as part of the dummy readings to be entered)	Refer to Step 3	Engagement:<br>Self-Awareness<br>+ Positive Behaviour Change
**19**	Create in-app calendar entry	Smartphone calendar sync	Functionality:<br>Feedback
**20**	Initiate daily medication reminder 06:30	Refer to Step 3	Ease of Use:<br>Reminders
**21**	Attempt to interact with other nominated users or support services (e.g. a helpline)	Refer to Step 3	Functionality:<br>Connection to Services
**22**	Attempt to change settings (e.g. fonts, colours, notifications)	Refer to Step 3	Engagement:<br>Customisation
**23**	Navigate to statistics screen and produce multiple views of data, e.g. weekly and/or monthly	Refer to Step 3	Information Management:<br>Statistics
**24**	Locate user prompts regarding lifestyle tips or behavioral advice based on user input, e.g. ramifications of medication overuse	Refer to Step 3	Engagement: Positive Behaviour Change
**25**	Locate option to connect app to peripheral device, e.g. Bluetooth monitor	Peripheral device and refer to Step 3	Engagement:<br>Use of Plug-ins
**26**	Is another health app required to supplement features not available in this app?	Refer to Step 3	Ease of Use:<br>Holistic Usability
**27**	Identify automated app features such as	Refer to Step 3	Ease of Use:

	pre-populated fields and appointment management		Automation
**28**	Identify password management option, privacy statement, cloud backup and data encryption	Refer to Step 3	Information Management: Privacy and Data Security
**29**	Observe extent of information provided for mobile experience	Refer to Step 3	Information Management: Quantity of Information
**30**	Recognize credible source of app, e.g. government lab logo, cites published research	Smartphone without internet connection	Information Management: Credibility
**31**	Turn off internet connection and test clinical data input and output displays	Smartphone without internet connection	Ease of Use: Offline Mode
